# Effect of Sow Intestinal Flora on the Formation of Endometritis

**DOI:** 10.3389/fvets.2021.663956

**Published:** 2021-06-18

**Authors:** Ling Zhang, Linkang Wang, Yimin Dai, Tianyu Tao, Jingqi Wang, Yunzheng Wu, Xiu Zeng, Jinhua Zhang

**Affiliations:** Institute of Animal Disease Prevent and Control, College of Animal Science and Technology, Jiangxi Agricultural University, Nanchang, China

**Keywords:** sow, endometritis, birth canal, intestinal flora, 16S rDNA gene

## Abstract

Endometritis is the main cause of decreased reproductive performance of sows, while one of the most important factors in the etiology of sow endometritis is an aberration of birth canal microbiota. Therefore, people began to pay attention to the microbiota structure and composition of the birth canal of sows with endometritis. Interestingly, we found that the risk of endometritis was increased in the sows with constipation in clinical practice, which may imply that the intestinal flora is related to the occurrence of endometritis. Therefore, understanding the relationship between birth canal microbiota and intestinal microbiota of the host has become exceptionally crucial. In this study, the microbiota of birth canal secretions and fresh feces of four healthy and four endometritis sows were analyzed via sequencing the V3 + V4 region of bacterial 16S ribosomal (rDNA) gene. The results showed a significant difference between endometritis and healthy sows birth canal flora in composition and abundance. Firmicutes (74.36%) and Proteobacteria were the most dominant phyla in birth canal microbiota of healthy sows. However, the majority of beneficial bacteria that belonging to Firmicutes phylum (e.g., *Lactobacillus* and *Enterococcus*) declined in endometritis sow. The abundance of *Porphyromonas, Clostridium sensu stricto 1, Streptococcus, Fusobacterium, Actinobacillus*, and *Bacteroides* increased significantly in the birth canal microbiota of endometritis sows. *Escherichia–Shigella* and *Bacteroides* were the common genera in the birth canal and intestinal flora of endometritis sows. The abundance of *Escherichia–Shigella* and *Bacteroides* in the intestines of sows suffering from endometritis were significantly increased than the intestinal microbiota of the healthy sows. We speculated that some intestinal bacteria (such as *Escherichia–Shigella* and *Bacteroides*) might be bound up with the onset of sow endometritis based on intestinal microbiota analysis in sows with endometritis and healthy sows. The above results can supply a theoretical basis to research the pathogenesis of endometritis and help others understand the relationship with the microbiota of sow's birth canal and gut.

## Introduction

Endometritis is a common and frequent reproductive system disease in the female domestic animal. It can lead to abnormal estrus, repeated infertility, or miscarriage in female animals, bringing enormous economic losses (1). Many previous studies have shown that birth canals flora plays a vital role in the formation of biological barriers that protect the health of the reproductive tract by the production of substances such as lactic acid, hydrogen peroxide, and bacteriocin (2). Recently, we found that the risk of endometritis was increased in the sows with constipation in clinical practice. Hence, understanding the gut and the birth canal flora is significant for preventing and treating endometritis of sow.

A growing evidence suggest that the intestinal microbiota affects numerous crucial physiological functions of the host, including immune system activation, metabolism, epithelial cell proliferation, and anti-infection (3). Nevertheless, there are few reports on the association between birth canal secretions and intestinal microbiota of sows. In this research, high-throughput sequencing of 16S ribosomal DNA (rDNA) gene was used to analyze the composition and differences in the vaginal secretions and intestinal bacterial communities of the sows in the health and endometritis. This is a novel attempt to reveal the pathogenesis of endometritis by the intestinal flora, providing new research ideas for the prevention and treatment of endometritis.

## Materials and Methods

### Experimental Design and Sample Collection

This research was approved by the Institutional Animal Care and Use Committee of Jiangxi Agricultural University and performed according to its guidelines (JXAULL-20190010). The clinical manifestations of sow endometritis mainly were increased body temperature, decreased feed intake, and thick yellow-brown discharge from the vagina. According to the above symptoms, four healthy sows and four sows suffering from endometritis were selected from a farm in Jingdezhen, Jiangxi, China. The vulvas of sows suffering from endometritis were bloodshot and puffy. Their vaginal secretions were multiplied, and many shed epithelial cells, white blood cells, etc., which can be observed under a microscope (Supplementary Figure 1). It is noteworthy that these eight sows came from the same unit; they had the same living environment and nutritional components of the diet. Vaginal secretion and fecal samples were collected from each sow. Moreover, there were no clinical abnormalities such as constipation and diarrhea in the intestines of sows with endometritis in this study. Fecal and vaginal secretions samples were put into sterilized containers immediately after sampling by a germ-free swab. They were kept at 4°C, transported to the laboratory, and then stored at −80°C until DNA extraction was performed.

### DNA Extraction and Sequencing

Total bacterial genomic DNA from samples was extracted using cetyltrimethyl ammonium bromide (CTAB)/sodium dodecyl sulfate (SDS) method and stored at −80°C for further experimentations. Sequencing was performed at Novogen Bioinformatics Technology Co., Ltd. (Beijing, China). Briefly, the V3 + V4 region of the bacterial 16S rDNA gene was amplified from the total extracted DNA using the 314F/806R primer set. All PCR reactions were performed with Phusion^®^ High Fidelity PCR Master Mix (New England Biolabs, Ipswich, MA, USA) with the following conditions: initial predenaturation of one cycle at 94°C for 3 min, followed by 38 cycles at 94°C for 45 s, at 55°C for 60 s, and at 72°C for 90 s, and a final extension step of one cycle of 72°C at 10 min. To separate and purify the PCR products, electrophoresis on 2% agarose gel and GeneJETTM Gel Extraction Kit (Thermo Scientific, Waltham, MA, USA) was conducted. Sequencing libraries were generated using Ion Plus Fragment Library Kit 48 rxns (Thermo Fisher, Waltham, MA, USA) following manufacturer's recommendations and were assessed on Qubit@2.0 Fluorometer (Thermo Fisher, Waltham, MA, USA) and then sequenced on an Ion S5TM XL platform. 16S rDNA sequencing data were saved in the European Nucleotide Archive (ENA) under the accession numbers ERS3526284–ERS3526299.

### Statistical Analysis of Microbial Community

Low-quality partial of the reads was sheared by using Cutadapt (V1.9.1, http://cutadapt.readthedocs.io/en/stable/). Then, the sample reads were split from the obtained reads according to a barcode, and the original reads were obtained by cutting off the initial quality control of the barcode and primer sequences. Quality filtering on the original reads was performed under specific filtering conditions to obtain the high-quality clean reads on the basis of the Cutadapt (V1.9.1) quality-controlled process. The reads were compared with the reference database (Silva Database, https://www.arb-silva.de/) using the UCHIME algorithm (UCHIME Algorithm, http://www.drive5.com/usearch/manual/uchime_algo.html) to detect and remove chimera sequences. Finally, we gained high-quality clean reads. Uparse software (Uparse v7.0.1001, http://drive5.com/uparse/) was used to analyze these sequences, and the sequences with ≥97% similarity were assigned to the same operational taxonomic units (OTUs). The representative sequences of each OTU were screened, and the Silva Database (https://www.arb-silva.de/) was used based on the Mothur algorithm to annotate taxonomic information.

Alpha and beta diversity analyses of the samples were performed with QIIME (Version 1.7.0) and displayed with R software (Version 2.15.3). Alpha diversity analysis included observed species, Chao1, Shannon, Simpson, ACE, and PD whole tree. The results were statistically analyzed using SPSS 22.0. Beta diversity included principal coordinates analysis. Principal coordinates analysis (PCoA) of the samples was performed based on weighted and unweighted Unifrac distance. According to the results of OUT clustering, the number of common and unique OTUs between different groups was analyzed using the Novomagic cloud platform and displayed with a Venn diagram. According to the results of species annotations, the top 20 and 30 species with the highest abundance of each group at the phylum and genus classification levels were selected to generate a columnar cumulative chart of relative abundance of species. The bacterial taxonomic differences represented between groups at the genus or higher taxonomy level were analyzed using LEfSe.

## Results

### Sequencing Results and Samples Diversity

A total of 16 samples [healthy vaginal secretion (HV, *N* = 4), healthy feces (HF, *N* = 4), endometritis vaginal secretion (EV, *N* = 4), and endometritis feces (EF, *N* = 4)] were collected from eight sows in a farm. Total DNA samples were extracted and sequenced on the Ion S5 XL platform. After cutting off the barcodes and primers and filtering low-quality reads and chimeras, a total of 1,212,768 high-quality sequences were acquired from all samples. These high-quality sequences were clustered into 7392 OTUs based on 97% similarity. Each sample contained 75,798 reads and 462 OTUs on average (see Supplementary Table 1). In this study, six alpha diversity measures were calculated including observed species (observed OTUs), Shannon, Simpson, Chao1, ACE, and PD whole tree (Supplementary Tables 2–5).

### Analysis of the Birth Canal Microbial Community of Endometritis Sows and Healthy Sows

**Figure 1:**
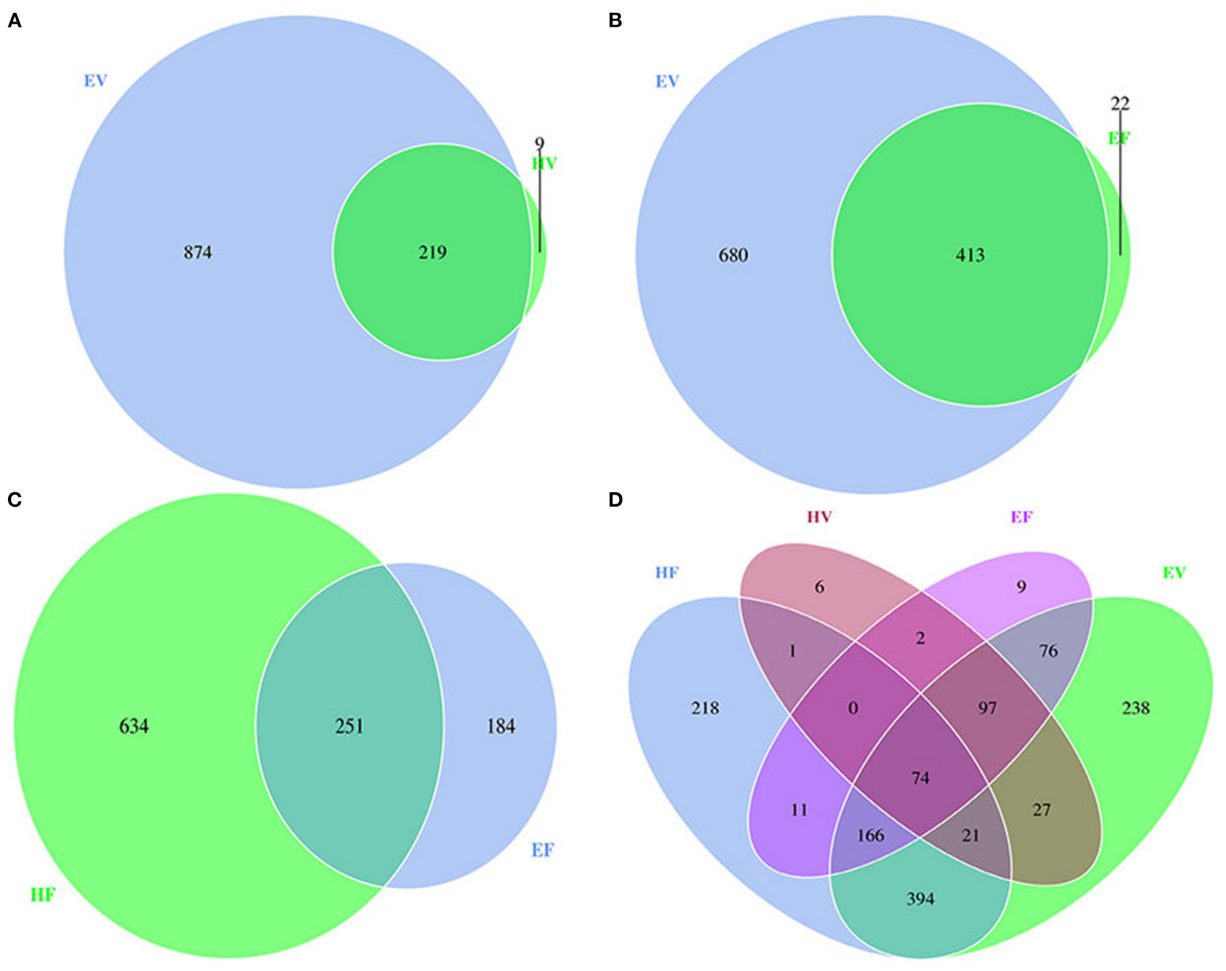
Compositions of the microbiota of the sows feces and vaginal secretions. **(A)** The number of operational taxonomic units (OTUs) shared in endometritis vaginal secretion (EV) and healthy vaginal secretion (HV) samples are shown in Venn diagrams; **(B)** the number of OTUs shared in EV and endometritis feces (EF) samples are shown in Venn diagrams; **(C)** the number of OTUs shared in healthy feces (HF) and EF samples are shown in Venn diagrams; **(D)** the number of OTUs shared in HF, HV, EF, and EV samples are shown in Venn diagrams.

**Figure 2:**
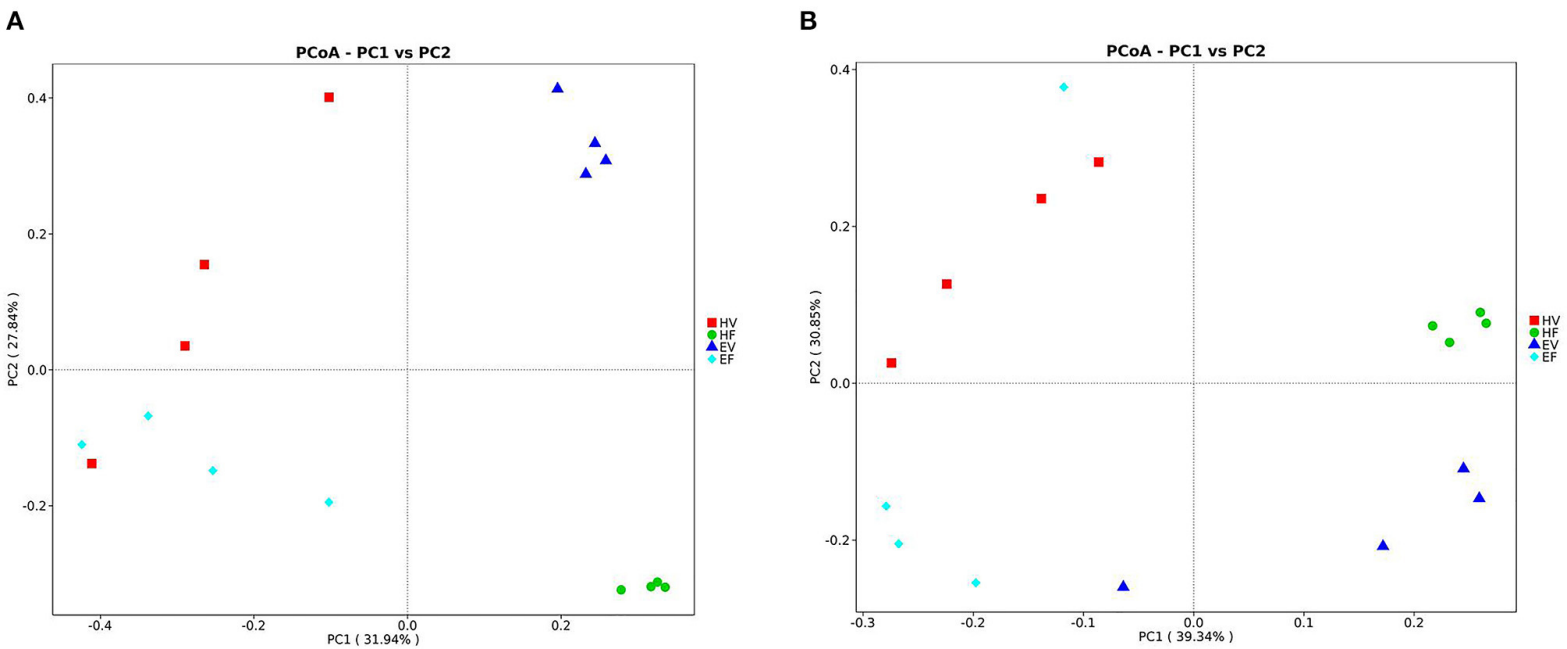
Principal coordinate analysis (PCoA) shows bacterial community structures based on Bray–Curtis distances. On the PCoA plot, each symbol represents one gut microbiome. **(A)** Unweighted UniFrac distance of the intestinal and vaginal sample microbiota; **(B)** weighted UniFrac distance of the intestinal and vaginal sample microbiota. The numbers of PC1 and PC2 show the percent variation explained by the PCoA plot.

Under the condition of 97% similarity, a total of 1,102 OTUs were observed in samples from HV and EV (Figure 1A). The microbiota of HV and EV samples shared 219 OTUs, with 874 and 9 OTUs uniquely identified from EV and HV samples, respectively (Figure 1A). The 874 OTUs unique to the EV group include 301 bacterial genera, the 9 OTUs unique to the HV group contain 3 bacterial genera, and the 219 OTUs shared by EV and HV contain 98 bacteria genera. Unlike the analysis of other microbiota, the diversity of HV microbiota was significantly lower than that of EV. In Figure 2, both the unweighted UniFrac distance (Figure 2A) and the weighted UniFrac distance (Figure 2B) could distinguish the significant difference in microbiota communities between EV and HV samples (Figure 2).

**Figure 3:**
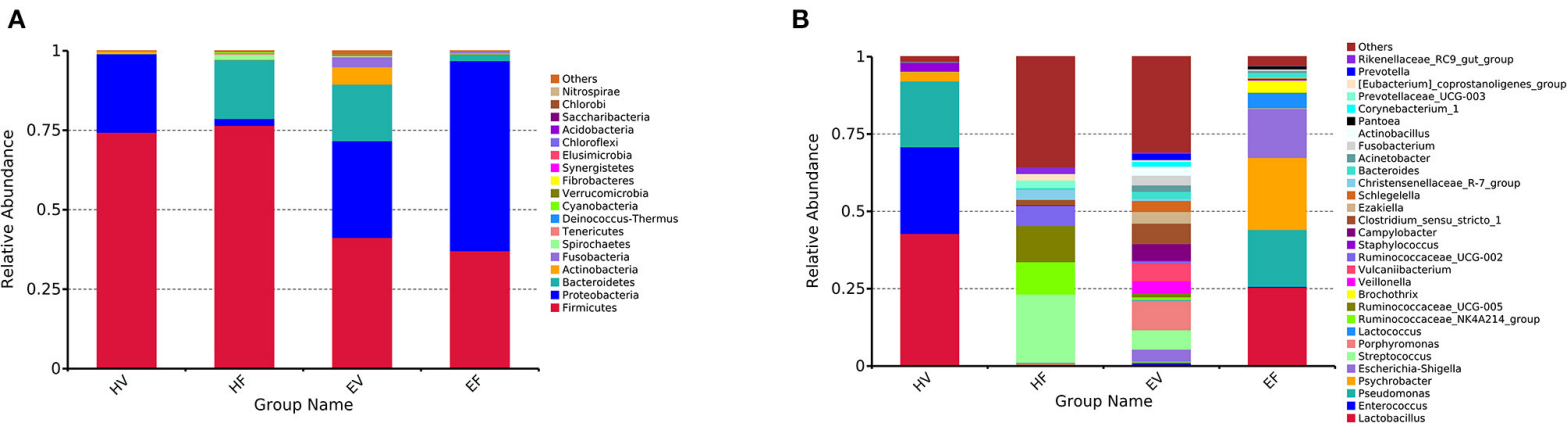
The overall compositions of the microbiota of HF, HV, EF, and EV. The overall compositions of the microbiota of the healthy feces (HF), healthy vaginal secretions (HV), endometritis feces (EF), and endometritis vaginal secretions (EV) were represented as bar plots at the **(A)** phylum level and the **(B)** genus level. Each bar represents the average relative abundance of each bacterial taxon within a group. The phylum-level shows the top 20 rich taxa, and the genus level shows the top 30 rich taxa.

**Figure 4:**
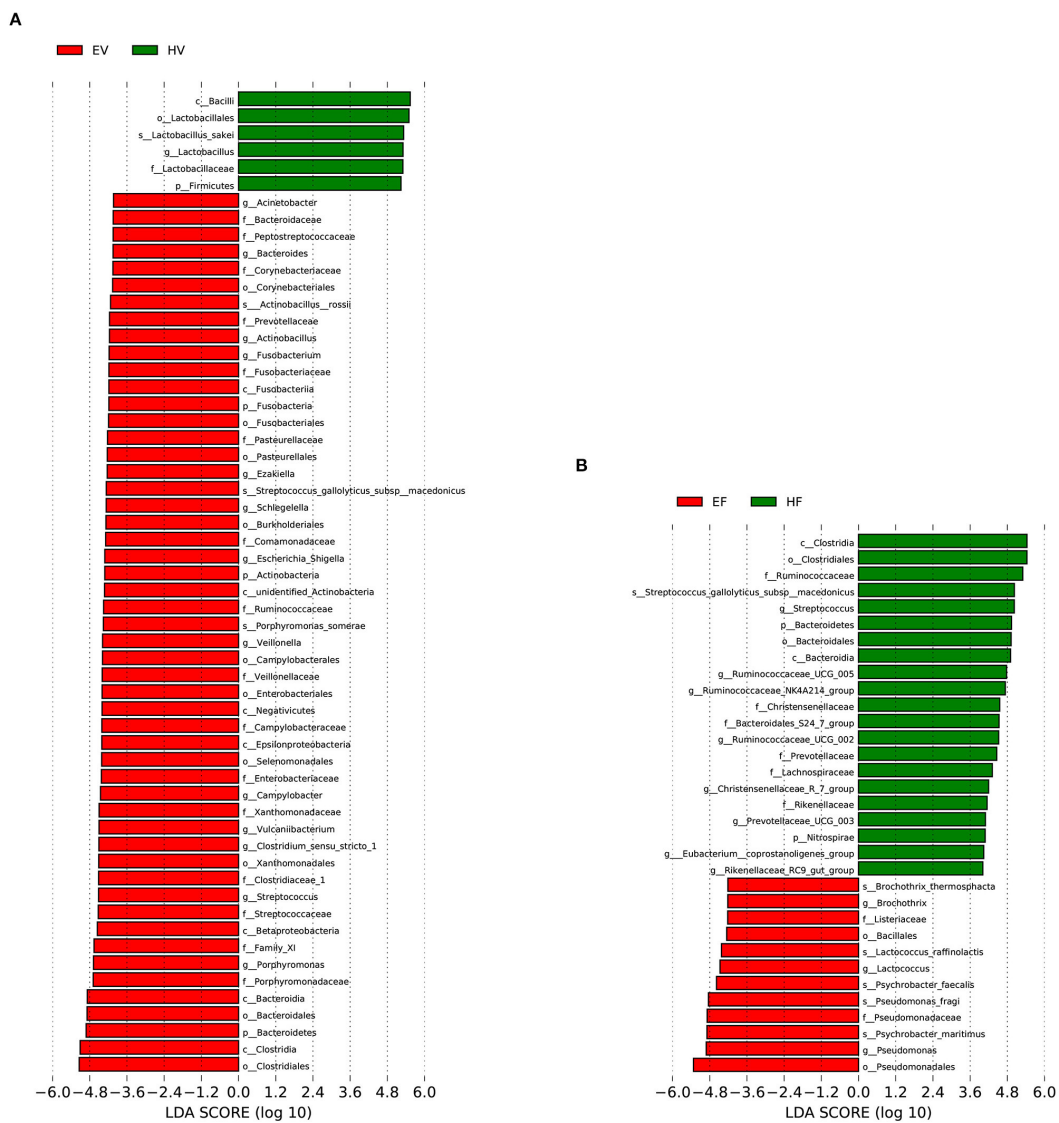
Bacterial taxa significantly differentiated between sample groups identified by linear discriminant analysis coupled with effect size (LEfSe) using the default parameters. **(A)** Different taxa between EV and HV samples; **(B)** different taxa between HF and EF samples.

Figure 3 showed the average relative abundance of the top 20 phyla and top 30 genera bacteria in the EV and HV samples. At the level of phylum, five predominant phyla were identified in the bacterial communities of EV and HV samples. On average, the relative abundance of these bacteria is over 1%. Firmicutes (41.26%) was the most predominant phylum in EV samples, followed by Proteobacteria (30.47%), Bacteroidetes (17.78%), Actinobacteria (5.48%), and Fusobacteria (3.17%) (Figure 3A). The most dominant bacteria in HV samples were Firmicutes (74.36%) and Proteobacteria (24.68%) (Figure 3A). The relative abundance of Firmicutes in HV was significantly higher than in EV (*p* < 0.05). A total of 337 genera were identified in the birth canal bacterial communities of endometritis sows compared to 100 genera in healthy sows. In EV samples, the most dominant bacterial genera included *Porphyromonas* (9.54%), *Clostridium_sensu_stricto_1* (6.66%), *Streptococcus* (6.26%), *Escherichia–Shigella* (3.84%), *Fusobacterium* (3.13%), and *Bacteroides* (2.30%) (Figure 3B). *Lactobacillus* (42.84%), *Enterococcus* (28.04%), *Pseudomonas* (21.27%), and *Psychrobacter* (3.02%) were the dominant genera in HV samples (Figure 3B). linear discriminant analysis effect size (LEfSe) analysis method was used to detect significant difference in bacterial taxa between EV and HV. The results showed that three bacterial species (*Actinobacillus rossi, Streptococcus gallolyticus* subsp. *macedonicus*, and *Porphyromonas somerae*) were significantly higher in EV samples than in HV samples. *Lactobacillus sakei* was significantly higher in HV samples compared with that in EV samples (Figure 4A and Supplementary Table 6).

### The Relationship Between the Birth Canal and Intestinal Flora of Endometritis Sows

A total of 1,115 OTUs were observed in samples from EV and EF (Figure 1B). The microbiota of EV and EF samples shared 413 OTUs, with 680 and 22 OTUs uniquely identified from EV and EF samples, respectively (Figure 1C). On the PCoA plot, the unweighted UniFrac distance (Figure 2A) could completely separate the EV samples from EF samples. However, the weighted UniFrac distance does not completely separate the EV sample from the EF sample (Figure 2B). This indicates that there is a certain similarity between the birth canal and intestinal flora of endometritis sows. Both Proteobacteria and Firmicutes have high relative abundance at the phylum levels in EV and EF samples (Supplementary Table 7).

At the genus levels, *Psychrobacter* (23.29%), *Pseudomonas* (18.38%), *Escherichia–Shigella* (15.91%), *Lactococcus* (4.91%), and *Bacteroides* (1.57%) were the other five dominant genera in EF samples (Figure 3B). In EV samples, *Porphyromonas* (9.54%), *Clostridium sensu stricto 1* (6.66%), *Streptococcus* (6.26%), *Escherichia–Shigella* (3.84%), *Fusobacterium* (3.13%), and *Bacteroides* (2.30%) were the predominant genera (Figure 3B). It is noteworthy that both *Escherichia–Shigella* and *Bacteroides* were common genera in EF and EV samples.

### Difference in Intestinal Community of Healthy Sows and Endometritis Sows

In the study, a total of 1,069 OTUs were observed in samples from HF and EF (Figure 1C). The microbiota of HF and EF samples shared 251 OTUs, with 634 and 184 OTUs uniquely identified from HF and EF samples, respectively. The community richness index (Chao1 and ACE) and community diversity index (Shannon) were significantly higher in HF samples than those in EF samples (*p* < 0.01) (Supplementary Table 4); only the Simpson Index did not show any significantly different (*p* < 0.05), indicating that both community richness and community diversity were dramatically higher in HF samples than in EF samples. Next, the unweighted UniFrac distance showed remarkable segregations of microbiota between HF and EF samples (Figure 2A). Similar discrimination was also observed, via weighted UniFrac distances, in the PCoA (Figure 2B), suggesting that the beta diversity of HF samples were also obviously higher than that of EF samples.

A total of 165 genera were identified in the intestine bacterial communities of endometritis sows compared to 211 genera in healthy sows. To identify the significant differences in gut bacteria between HF and EF samples, we compared the relative abundance of gut bacteria in HF and EF samples. The result indicated that the relative abundance of five genera (*Lactobacillus, Pseudomonas, Psychrobacter, Escherichia–Shigella*, and *Bacteroides*) increased obviously in the EF samples, although the difference is not significant (Supplementary Table 8). LEfSe analysis also revealed that *Lactococcus raffinolactis, Psychrobacter faecalis, Pseudomonas fragi*, and *Psychrobacter maritimus* were significantly higher in EF samples compared with HF samples (Figure 4B). In brief, compared with HV samples, there were 874 (238 + 76 + 394 + 166) unique OTUs in EV samples, of which 242 (76 + 166) OTUs were shared with EF; while among these 242 OTUs, 166 OTUs were shared with HF, and only 76 OTUs were shared with EF. Seventy-six OTUs were the key in understanding the relationship between the occurrence of sow endometritis and its intestinal flora (Figure 1D). We found that there were 5 bacterial phyla, mainly including Firmicutes and Bacteroidetes, and 46 bacterial genera in 76 OUTs; *Bacteroides* was the most abundant genus.

## DISCUSSION

### Differences Between the Birth Canal Microbiota of Endometritis Sows and Healthy Sows

Despite documented evidence indicating that birth canal flora has a key function in the etiology of endometritis, the structure and composition of the birth canal microbiota in endometritis sows are still not well-elucidated. At the phylum level, the relative abundance of Firmicutes was the highest in the EV samples. Bacteroidetes, Actinobacteria, and Fusobacteria were only present in the EV samples when compared with HV samples. At the genus level, the relative abundance of *Porphyromonas, Clostridium sensu stricto 1, Streptococcus, Fusobacterium, Escherichia–Shigella, Actinobacillus*, and *Bacteroides* were remarkably higher in EV samples than those in HV samples. A growing evidence suggest that *Porphyromonas, Clostridium sensu stricto 1, Streptococcus, Fusobacterium, Actinobacillus*, and *Bacteroides* closely correlated with diseases in the animal. For instance, *Porphyromonas* species resulted in particularly significant endometrial cancer (4). Correlation studies showed that the messenger RNA (mRNA) expression of interleukin 1β (IL-1β) and tumor necrosis factor alpha (TNF-α) positively correlated with the enrichments in *Clostridium sensu stricto 1* in the colon mucosal of sheep; this enrichment eventually leads to inflammation of the colonic epithelium in sheep (5). Xiaojing Xia et al. reported that the pathogenic protein secreted by *Streptococcus* could escape host phagocytosis and complement-mediated immune destruction leading to the onset in the body (6). Moreover, Wang et al. reported that *Clostridium sensu stricto 1, Fusobacterium*, and *Bacteroides* are more abundant in the vagina of endometritis sows compared to healthy sows (7). Similar results have been obtained in studies of human bacterial vaginosis, compared with healthy women; Bacteroidetes, Actinobacteria, and Fusobacteria were more abundant in bacterial vaginosis in women (8). These investigations provided strong evidence for our findings that changes in the birth canal flora may be related to the occurrence of endometritis.

Another apparent difference in EV samples was the decreased relative abundance of Firmicutes members, including *Lactobacillus* and *Enterococcus*. We observed statistical predominance of *Lactobacillus* in 9 OTUs unique to HV samples. Firmicutes was reported to have the highest abundant phenotype in birth canals samples of the healthy sows (7), and a few members of this phylum are considered to adjust to systemic immune responses (9). Therefore, these beneficial bacteria may regulate bacteria balance, inhibit conditional pathogens, and prevent colonization of pathogenic microorganisms. For instance, *Lactobacillus* are generally studied as probiotic agents, affecting pathogenicity of opportunistic pathogens and host immune regulation (10). *L. sakei* releases spherical membrane vesicles (MVs) through its cell wall components by activating host TLR2 signals, thereby enhancing the production of IgA, preventing the incursion of pathogenic microorganisms, and regulating the composition of intestines microbiota (11). In this study, *Lactobacillus* and *Enterococcus* were found at lower levels in EV samples, which are consistent with the previous studies.

### Differences Between the Intestines Microbiota of Endometritis and Healthy Sows

The imbalances of microbiota and abnormal immune responses to intestine bacteria can destroy intestinal and host homeostasis (12). Bacterial exogenous infection is one of the important causes of endometritis. In clinical practice, we found that sows with constipation have a higher risk of endometritis. To understand the influence of intestinal flora on endometritis, we compared the microbial composition of EF and HF. In this study, we observed that the diversity of the intestinal flora of sows with endometritis were significantly reduced. A previous study reported that the higher the diversity of intestinal flora, the stronger is its ability to maintain the balance of intestinal flora (13). The results showed that: *Lactobacillus, Psychrobacter, Pseudomonas*, and *Escherichia–Shigella* were more abundant in EF samples, compared with HF samples. In the study of Zhao, the amount of *Lactobacillus* was increased in the mouse with spleen-deficiency constipation (14). *Pseudomonas* and *Psychrobacter* have been confirmed to be related to certain animal diseases. For instance, some scholars have reported that *Pseudomonas* can be an oral and tracheal pathogen in premature infants (15). The members of genus *Psychrobacter* are considered to be opportunistic pathogens, as they are occasionally isolated from infected animals and human patients (16). The above findings suggest that the homeostasis of the intestinal flora of sows with endometritis had changed, and whether this change is necessarily related to constipation requires further research.

### Effect of Sow Birth Canals and Gastrointestinal Flora on Endometritis

The birth canal and gastrointestinal tract of mammals are highly complex ecosystems that play an important role in animal health and disease. In this study, we found that the birth canal and gastrointestinal flora of sows with endometritis were different from that of healthy sows. Firmicutes, Proteobacteria, and Bacteroidetes are the main bacterial phyla in the birth canals and gastrointestinal flora of endometritis sows, which is similar to the result by Koh et al. (17). At the genus level, *Escherichia–Shigella* and *Bacteroides* were bacterial genus shared in EF and EV samples. Wang et al. found that the abundance of *Escherichia–Shigella* and *Bacteroides* in sows with endometritis was higher than that in healthy sows (7). The abundance of *Escherichia–Shigella* was positively correlated with ulcerative colitis (18). A study by Wang et al. reported that *Bacteroides* could lead to an endogenous infection when the immune system or intestinal microbiota is dysfunctional (19).

We speculate about that the intestinal microbiota (*Escherichia–Shigella* and *Bacteroides*) of the sow may affect the balance of the flora of the birth canal and promote the growth and reproduction of opportunistic pathogens, leading to endometritis. Specifically, how it affects requires further research to clarify.

In addition, the high abundance of *Bacteroides* found in cows with endometritis also indicated that *Bacteroides* were highly associated with uterine disease (20). Some scholars have found that the first colonization flora of humans originates from maternal microorganisms (21). In our previous study, the bacteria *E. coli, Shigella*, and *Clostridium* existing in endometritis sows were also the main dominant bacteria in the intestines of a group of diarrhea piglets (22). The *Lactobacillus*, which is more abundant in the birth canal of healthy sows, has been reported to have the effect of alleviating diarrhea in piglets (23). The bacteriocin secreted by *Lactobacillus* can promote the absorption of intestinal fluid and reduce the secretion of intestinal fluid by activating phosphodiesterase activity and reducing cAMP and cGMP levels (24). These indicate that the sow's birth canal microbiota may be related to the health of the piglets.

Taken together, the imbalance of sow intestinal flora may affect the balance of the birth canal microbiota and lead to endometritis, while the vertical transmission of birth canal microbes will affect the health of piglets. Specifically, the way how sow gut microbiota affects its birth canal and piglet microbiota (or the regulatory mechanism of the impact) needs further study to clarify.

In conclusion, our study unveiled differences in birth canals microbiota between endometritis and healthy sows and described the correlation between the birth canal and the gut microbiota of the endometritis sow. The results showed that *Porphyromonas, Clostridium sensu stricto 1, Streptococcus, Fusobacterium, Escherichia-Shigella*, and *Bacteroides* might be related to the occurrence of sow endometritis. Among them, *Escherichia–Shigella* and *Bacteroides* may be related to the intestinal flora of endometritis sows. Simultaneously, we have also found that a decrease in the abundance of *Lactobacillus* could lead to a diversity increase in the flora of the birth canal, and the latter has the risk of causing endometritis. These findings can provide a theoretical basis to study endometritis, the sow's birth canal and gut microbiota, and will be helpful to establish an effective strategy to reduce postpartum disease generating in sows.

## Data Availability Statement

The original contributions presented in the study are included in the article/Supplementary Material, further inquiries can be directed to the corresponding author/s.

## Ethics Statement

The animal study was reviewed and approved by Animal Experiment Welfare Ethics Committee of Jiangxi Agricultural University.

## Author Contributions

JZ and YD designed the study. LW, YW, and XZ collected the sows fecal and vaginal secretions samples. LW and YW performed the experiments. LW, JW, and XZ analyzed the data. LZ, LW, TT, and JZ wrote and revised the manuscript. All authors read and approved the final manuscript.

## Conflict of Interest

The authors declare that the research was conducted in the absence of any commercial or financial relationships that could be construed as a potential conflict of interest.
