# Supplementary material for: Effect of Sow Intestinal Flora on the Formation of Endometritis
Source: Front Vet Sci. 2021 Jun 18;8:663956. doi: 10.3389/fvets.2021.663956 (PMC8249707; doi:10.3389/fvets.2021.663956)
Supplement: Supplementary file 1 [file Data_Sheet_1.ZIP › Supplementary material/Supplementary material/Supplementary Table S1.docx]

**Supplementary Table S1** OTU table summary

| Sample name | Health status | Sample Type | Total tag | OTUs |
| --- | --- | --- | --- | --- |
| HV1  HV2  HV3  HV4  HF1  HF2  HF3  HF4  EV1  EV2  EV3  EV4  EF1  EF2  EF3  EF4 | Health  Health  Health  Health  Health  Health  Health  Health  Endometritis  Endometritis  Endometritis  Endometritis Endometritis  Endometritis  Endometritis  Endometritis | Vaginal secretion  Vaginal secretion  Vaginal secretion  Vaginal secretion  Feces  Feces  Feces  Feces  Vaginal secretion  Vaginal secretion  Vaginal secretion  Vaginal secretion  Feces  Feces  Feces  Feces | 80735  80057  80108  80699  65133  67184  82538  85959  45425  62538  80237  80076  94425  72633  86024  68989 | 122  158  161  172  607  705  747  671  585  797  876  756  109  216  311  401 |
