# Supplementary material for: Effect of Sow Intestinal Flora on the Formation of Endometritis
Source: Front Vet Sci. 2021 Jun 18;8:663956. doi: 10.3389/fvets.2021.663956 (PMC8249707; doi:10.3389/fvets.2021.663956)
Supplement: Supplementary file 1 [file Data_Sheet_1.ZIP › Supplementary material/Supplementary material/Supplementary Table S3.docx]

**Supplementary Table S3 |** Differences in vaginal secretions and fecal microbiota alpha diversity in endometritis sows

| Item | EV | EF | P Value | |
| --- | --- | --- | --- | --- |
| Alpha diversity  Observed-species  Shannon  Simpson  Chao1  ACE  PD-whole-tree | 706.50±87.03  6.29±0.50  0.97±0.01  879.64±90.94  797.69±81.57  94.96±14.51 | 199.50±107.84  1.96±1.26  0.51±0.33  250.55±122.11  264.85±113.24  18.19±7.47 | | 0.001**  0.001**  0.048*  0.020*  0.001**  0.001** |

The data were expressed as the mean values ± standard deviation (SD)

The P values were determined using Welch’s t test (* P < 0.05; ** P < 0.01)
