# Supplementary material for: Effect of Sow Intestinal Flora on the Formation of Endometritis
Source: Front Vet Sci. 2021 Jun 18;8:663956. doi: 10.3389/fvets.2021.663956 (PMC8249707; doi:10.3389/fvets.2021.663956)
Supplement: Supplementary file 1 [file Data_Sheet_1.ZIP › Supplementary material/Supplementary material/Supplementary Table S4.docx]

**Supplementary Table S4 |** Differences in the feces microbiota alpha diversity of healthy and endometritis sows

| Item | HF | EF | P Value |
| --- | --- | --- | --- |
| Alpha diversity  Observed-species  Shannon  Simpson  Chao1  ACE  PD-whole-tree | 631.50±61.02  5.85±0.33  0.92±0.05  694.46±84.70  705.25±89.79  38.88±2.45 | 199.50±107.84  1.96±1.26  0.51±0.33  250.55±122.11  264.85±113.24  18.19±7.47 | 0.001**  0.007**  0.089  0.006**  0.001**  0.008** |

The data were expressed as the mean values ± standard deviation (SD)

The P values were determined using Welch’s t test (* P < 0.05; ** P < 0.01)
