# Supplementary material for: Effect of Sow Intestinal Flora on the Formation of Endometritis
Source: Front Vet Sci. 2021 Jun 18;8:663956. doi: 10.3389/fvets.2021.663956 (PMC8249707; doi:10.3389/fvets.2021.663956)
Supplement: Supplementary file 1 [file Data_Sheet_1.ZIP › Supplementary material/Supplementary material/Supplementary Table S5.docx]

**Supplementary Table S5 |** Differences in vaginal secretions and fecal microbiota alpha diversity in healthy sows

| Item | HV | | HF | | P Value |
| --- | --- | --- | --- | --- | --- |
| Alpha diversity |  |  | |  | |
| Observed-species  Shannon  Simpson  Chao1  ACE  PD-whole-tree | 112.00±17.66  1.27±0.79  0.41±0.29  178.92±40.91  204.45±49.93  23.20±12.46 | | 631.50±61.02  5.85±0.33  0.92±0.05  694.46±84.70  705.25±89.79  38.88±2.45 | | 0.000**  0.000**  0.037*  0.002**  0.000**  0.084 |

The data were expressed as the mean values ± standard deviation (SD)

The P values were determined using Welch’s t test (* P < 0.05; ** P < 0.01)
