# Supplementary material for: Effect of Sow Intestinal Flora on the Formation of Endometritis
Source: Front Vet Sci. 2021 Jun 18;8:663956. doi: 10.3389/fvets.2021.663956 (PMC8249707; doi:10.3389/fvets.2021.663956)
Supplement: Supplementary file 1 [file Data_Sheet_1.ZIP › Supplementary material/Supplementary material/Supplementary Table S6.docx]

**Supplementary Table S6 |** Differences in the vaginal secretions microbiota of the healthy sows and the endomentritis sows

| Taxon | EV | HV | P value |
| --- | --- | --- | --- |
| Phylum(%)  *Firmicutes*  *Proteobacteria*  *Bacteroidetes*  *Actinobacteria*  *Fusobacteria*  Genus(%)  *Lactobacillus*  *Enterococcus*  *Pseudomonas*  *Psychrobacter*  *Staphylococcus*  *Porphyromonas*  *Clostridium-sensu-stricto-1*  *Streptococcus*  *Vulcaniibacterium*  *Campylobacter*  *Veillonella*  *Escherichia-Shigella*  *Ezakiella*  *Schlegelella*  *Fusobacterium*  *Actinobacillus*  *Bacteroides*  *Prevotella*  *Methyloversatilis*  *Terrisporobacter*  *Corynebacterium_1*  *Anaerococcus*  *Murdochiella*  *Gallicola*  *Ruminococcaceae_UCG-005* | 41.26±0.03  30.47±0.14  17.78±0.09  5.48±0.03  3.17±0.01  0.57±0.00  0.44±0.00  0.35±0.00  0.26±0.00  0.29±0.00  9.54±0.07  6.66±0.03  6.26±0.02  5.88±0.05  5.24±0.05  3.98±0.07  3.84±0.03  3.74±0.03  3.59±0.03  3.13±0.01  2.92±0.01  2.30±0.01  2.07±0.01  1.73±0.01  1.62±0.01  1.52±0.02  1.40±0.01  1.39±0.01  1.35±0.01  1.23±0.01 | 74.36±0.23  24.68±0.23  0.07±0.00  0.84±0.02  0.01±0.00  42.84±0.35  28.04±0.47  21.27±0.25  3.02±0.06  2.91±0.06  0.01±0.00  0.05±0.00  0.05±0.00  0.01±0.00  0.01±0.00  0.01±0.00  0.02±0.00  0.01±0.00  0.00±0.00  0.01±0.00  0.00±0.00  0.02±0.00  0.00±0.00  0.00±0.00  0.04±0.00  0.01±0.00  0.00±0.00  0.00±0.00  0.00±0.00  0.00±0.00 | 0.031*  0.685  0.029*  0.038*  0.010*  0.092  0.325  0.196  0.402  0.425  0.075  0.015*  0.011*  0.118  0.115  0.328  0.105  0.034*  0.106  0.012*  0.009**  0.036*  0.032*  0.093  0.024*  0.158  0.027*  0.082  0.120  0.056 |

The data were expressed as the mean values ± standard deviation (SD)

The P values were determined using Welch’s t test (* P < 0.05; ** P < 0.01)
