# Supplementary material for: Effect of Sow Intestinal Flora on the Formation of Endometritis
Source: Front Vet Sci. 2021 Jun 18;8:663956. doi: 10.3389/fvets.2021.663956 (PMC8249707; doi:10.3389/fvets.2021.663956)
Supplement: Supplementary file 1 [file Data_Sheet_1.ZIP › Supplementary material/Supplementary material/Supplementary Table S7.docx]

**Supplementary Table S7 |** Differences in the vaginal secretions and the fecal microbiota of the endomentritis sows

| Taxon | EV | EF | P value |
| --- | --- | --- | --- |
| Phylum(%)  *Firmicutes*  *Proteobacteria*  *Bacteroidetes*  *Actinobacteria*  *Fusobacteria*  Genus(%)  *Lactobacillus*  *Pseudomonas*  *Psychrobacter*  *Escherichia-Shigella*  *Lactococcus*  *Brochothrix*  *Bacteroides*  *Porphyromonas*  *Clostridium_sensu_stricto_1*  *Streptococcus*  *Vulcaniibacterium*  *Campylobacter*  *Veillonella*  *Ezakiella*  *Schlegelella*  *Fusobacterium*  *Actinobacillus*  *Acinetobacter*  *Prevotella*  *Methyloversatilis*  *Anaerococcus* | 41.26±0.07  30.47±0.14  17.78±0.09  5.48±0.03  3.17±0.01  0.57±0.00  0.35±0.00  0.26±0.00  3.84±0.03  0.19±0.00  0.00±0.00  2.30±0.01  9.54±0.07  6.66±0.03  6.26±0.02  5.88±0.05  5.24±0.05  3.98±0.07  3.74±0.03  3.59±0.03  3.13±0.01  2.92±0.01  2.09±0.02  2.07±0.01  1.73±0.01  1.40±0.01 | 37.07±0.42  59.84±0.39  2.11±0.02  0.41±0.01  0.49±0.00  25.47±0.49  18.38±0.36  23.29±0.33  15.91±0.31  4.91±0.08  3.84±0.08  1.57±0.02  0.10±0.00  0.29±0.00  0.10±0.00  0.05±0.00  0.04±0.00  0.00±0.00  0.13±0.00  0.03±0.00  0.48±0.00  0.03±0.00  0.61±0.01  0.01±0.00  0.02±0.00  0.23±0.00 | 0.855  0.238  0.036*  0.035*  0.010*  0.382  0.392  0.260  0.495  0.301  0.385  0.582  0.077  0.016*  0.011*  0.120  0.117  0.328  0.078  0.107  0.012*  0.009**  0.214  0.070  0.095  0.053 |

The data were expressed as the mean values ± standard deviation (SD)

The P values were determined using Welch’s t test (* P < 0.05; ** P < 0.01)
