# Supplementary material for: Effect of Sow Intestinal Flora on the Formation of Endometritis
Source: Front Vet Sci. 2021 Jun 18;8:663956. doi: 10.3389/fvets.2021.663956 (PMC8249707; doi:10.3389/fvets.2021.663956)
Supplement: Supplementary file 1 [file Data_Sheet_1.ZIP › Supplementary material/Supplementary material/Supplementary Table S8.docx]

**Supplementary Table S8 |** Differences in the fecal microbiota of the healthy sows and the endomentritis sows

| Taxon | HF | EF | P value |
| --- | --- | --- | --- |
| Phylum(%)  *Firmicutes*  *Proteobacteria*  *Bacteroidetes*  *Spirochaetes*  Genus(%)  *streptococcus*  *Ruminococcaceae-NK4A214-group*  *Ruminococcaceae-UGG-005*  *Ruminococcaceae-UGG-002*  *Lactobacillus*  *Pseudomonas*  *Psychrobacter*  *Escherichia-Shigella*  *Lactococcus*  *Brochothrix*  *Christensenellaceae_R-7_group*  *Prevotellaceae_UCG-003*  *Prevotellaceae_NK3B31_group*  *Ruminococcaceae_UCG-010*  *Rikenellaceae_RC9_gut_group*  *Clostridium_sensu_stricto_1*  *Treponema_2*  *Bacteroides*  *Lachnospiraceae_XPB1014_group*  *Family_XIII_AD3011_group* | 76.53±0.07  2.18±0.01  18.52±0.07  1.54±0.01  22.04±0.15  10.47±0.06  11.68±0.03  6.49±0.04  0.23±0.00  0.01±0.00  0.46±0.01  0.50±0.01  0.00±0.00  0.00±0.00  3.20±0.02  2.39±0.01  1.15±0.00  1.37±0.01  2.19±0.01  1.74±0.01  1.52±0.01  0.63±0.00  1.34±0.00  1.03±0.00 | 37.07±0.42  59.84±0.39  2.11±0.02  0.02±0.00  0.10±0.00  0.06±0.00  0.06±0.00  0.08±0.00  25.47±0.49  18.38±0.36  23.29±0.33  15.91±0.31  4.91±0.08  3.84±0.08  0.08±0.00  0.00±0.00  0.00±0.00  0.01±0.00  0.03±0.00  0.29±0.00  0.02±0.00  1.57±0.02  0.02±0.00  0.03±0.00 | 0.154  0.061  0.013*  0.047*  0.025*  0.009**  0.000**  0.013*  0.376  0.384  0.264  0.395  0.032*  0.349  0.021*  0.007**  0.001**  0.042*  0.011*  0.023*  0.017*  0.415  0.001**  0.005** |

The data were expressed as the mean values ± standard deviation (SD)

The P values were determined using Welch’s t test (* P < 0.05; ** P < 0.01)
