# Supplementary material for: Effect of Sow Intestinal Flora on the Formation of Endometritis
Source: Front Vet Sci. 2021 Jun 18;8:663956. doi: 10.3389/fvets.2021.663956 (PMC8249707; doi:10.3389/fvets.2021.663956)
Supplement: Supplementary file 1 [file Data_Sheet_1.ZIP › Supplementary material/Supplementary material/Supplementary Table S9.docx]

**Supplementary Table S9 |** Differences in the vaginal secretions and the fecal microbiota of the healthy sows

| Taxon | HV | HF | P value |
| --- | --- | --- | --- |
| Phylum(%)  *Firmicutes*  *Proteobacteria*  *Bacteroidetes*  *Spirochaetes*  Genus(%)  *Lactobacillus*  *Enterococcus*  *Pseudomonas*  *Psychrobacter*  *Staphylococcus*  *Streptococcus*  *Ruminococcaceae_NK4A214*  *Ruminococcaceae_UCG-005*  *Ruminococcaceae_UCG-002*  *Christensenellaceae_R-7_group*  *Prevotellaceae_UCG-003*  *Rikenellaceae_RC9_gut_group*  *Clostridium_sensu_stricto_1*  *Treponema_2*  *Ruminococcaceae_UCG-010*  *Lachnospiraceae_XPB1014_group*  *Prevotellaceae_NK3B31_group*  *Family_XIII_AD3011_group* | 74.36±0.23  24.68±0.23  0.07±0.00  0.01±0.00  42.84±0.34  28.04±0.47  21.27±0.025  3.02±0.06  2.91±0.06  0.05±0.00  0.00±0.00  0.00±0.00  0.00±0.00  0.01±0.00  0.00±0.00  0.00±0.00  0.05±0.00  0.01±0.00  0.00±0.00  0.00±0.00  0.00±0.00  0.00±0.00 | 76.53±0.0  2.18±0.01  18.52±0.07  1.54±0.01  0.23±0.00  0.01±0.00  0.01±0.00  0.46±0.01  0.00±0.00  22.04±0.15  10.47±0.06  11.68±0.03  6.49±0.04  3.20±0.02  2.39±0.01  2.19±0.01  1.74±0.00  1.52±0.01  1.37±0.01  1.34±0.00  1.15±0.00  1.04±0.00 | 0.864  0.145  0.013*  0.046*  0.090  0.318  0.191  0.437  0.346  0.059  0.032*  0.006**  0.039*  0.050  0.007**  0.010*  0.033*  0.046*  0.081  0.007**  0.007**  0.020* |

The data were expressed as the mean values ± standard deviation (SD)

The P values were determined using Welch’s t test (* P < 0.05; ** P < 0.01)
